# Homozygous deletion of *CDKN2A* by fluorescence in situ hybridization is prognostic in grade 4, but not grade 2 or 3, IDH-mutant astrocytomas

**DOI:** 10.1186/s40478-020-01044-y

**Published:** 2020-10-20

**Authors:** Daniel F. Marker, Thomas M. Pearce

**Affiliations:** Department of Pathology, Division of Neuropathology, University of Pittsburgh Medical Center, S701 Scaife Hall, 3550 Terrace Street, Pittsburgh, PA 15261 USA

**Keywords:** CDKN2A, IDH, Astrocytoma, FISH

## Abstract

IDH-mutant astrocytomas have a more indolent natural history and better prognosis than their IDH-wild type counterparts, but are still graded according to schemes developed prior to the recognition of this type of neoplasm as a distinct entity. Homozygous deletion of *CDKN2A* has been proposed as a molecular correlate of aggressive behavior in these tumors, and may be incorporated into future grading systems in an effort to improve prognostic stratification. Fluorescence in situ hybridization (FISH) is a common ancillary testing modality used to assess *CDKN2A* status, but the specifics of how to best interpret FISH results for prognostication of gliomas have not been clearly defined in the literature. To address this issue, we performed a retrospective analysis of prospectively collected *CDKN2A* FISH data from 108 primary and 43 recurrent IDH-mutant astrocytomas diagnosed between 2007–2020 at the University of Pittsburgh Medical Center. High level *CDKN2A* homozygous deletion was rare in primary tumors and was identified more frequently in recurrent tumors. Multivariate Cox Proportional-Hazards analysis demonstrated that histologic grade and *CDKN2A* status are independent predictors of survival, and the prognostic value of *CDKN2A* is maximized by applying a threshold of ≥ 30% of tumor cells with homozygous deletion by FISH to define a positive result. At this threshold, *CDKN2A* deletion significantly stratified survival of histologic grade 4 tumors, but grade 2 and 3 tumors rarely exceeded this cutoff value and did not show worse survival. Lower thresholds identified additional lower grade tumors, but were not prognostically useful. Compared to prior studies, the lack of prognostic significance of *CDKN2A* homozygous deletion by FISH in grade 2–3 IDH-mutant astrocytomas may reflect differences in cohort populations or technical differences between testing modalities. Definitive criteria for determining *CDKN2A* homozygous deletion by various methodologies will be critical if this is to be included in future grading schemes.

## Introduction

Incorporation of somatic molecular alterations into the classification and grading of brain tumors continues to drive an ongoing revolution in the field of neuro-oncology. Among diffusely infiltrating gliomas, the mutational status of isocitrate dehydrogenase (*IDH1* or *IDH2* genes) is a fundamental determinant of biologic behavior, which, in combination with additional molecular features, is now a defining criterion of the diagnostic category of IDH-mutant (IDHm) astrocytoma [1]. Segregating IDHm astrocytic tumors from their more aggressive IDH-wild type (IDHwt) counterparts was a major advance in our understanding of diffuse glioma biology and prognostication. Current WHO grading criteria, however, do not differentiate IDHm from IDHwt astrocytomas and have not yet been updated to reflect the differing clinical behaviors of these diagnostic entities. Additionally, some of the morphologic features used to grade IDHwt tumors do a poor job of stratifying IDHm astrocytoma survival, particularly mitotic activity [2–4]. To address these shortcomings, a number of groups have investigated molecular correlates of aggressive behavior in IDHm astrocytomas [5–11]. Based on these studies, homozygous deletion of the *CDKN2A* gene, which encodes the cell-cycle regulators p16INK4A and p14ARF, has emerged as a leading candidate molecular marker of high-grade behavior in these tumors [3, 7, 10–12].

Given the likelihood that *CDKN2A* status may be incorporated into future grading systems for IDHm astrocytomas, we sought to understand how such a change might impact the practice of neuropathology at our institution. In particular, we wished to define how best to interpret data from fluorescence in situ hybridization (FISH) studies, since the influential studies showing prognostic utility of *CDKN2A* loss [7, 10] have largely utilized array-based techniques that are not yet readily accessible to many institutions world-wide. Array based methods rely on algorithmically-defined thresholds to binarize the presence or absence of copy number loss, while the percentage of cells showing homozygous deletion by FISH is a continuous variable. FISH provides a direct visualization of the status of the genetic locus of interest and readily distinguishes true homozygous deletion from hemizygous loss or complex copy number change. FISH testing is advantageous in the setting of diffusely infiltrative tumors with highly variable degrees of tumor cellularity relative to background non-neoplastic parenchyma, since the sensitivity of sequencing- and array-based techniques for accurately detecting homozygous deletion suffers in this situation. The choice of where to set the clinical cutoff for defining tumor wide *CDKN2A* deletion based on the proportion of tumor cells with homozygous deletion will be critically important if *CDKN2A* status is to be used for tumor grading and therapeutic decision-making [3, 12].

To address these issues, we leveraged our decades-long institutional experience with molecular testing of gliomas. For many years prior to the application of routine next-generation sequencing (NGS) of brain tumors, *CDKN2A* status was evaluated by FISH as part of the standard evaluation of newly-diagnosed and recurrent gliomas at our institution. Our anecdotal impressions from this period agreed with the developing literature that high levels of *CDKN2A* homozygous deletion portended poor outcome, but suggested that tumors with low-level homozygous deletion were not uniformly aggressive. In addition, cases of recurrent tumors that had developed new *CDKN2A* deletion compared to the original resection specimen led us to hypothesize that this molecular marker might have different prognostic implications at the time of recurrence compared to initial presentation. To move beyond anecdotes, we systematically analyzed our institutional cohort of patients with IDHm astrocytomas and *CDKN2A* FISH analysis in order to better define the relationship between percentage of cells with homozygous *CDKN2A* deletion, histologic grade, and patient survival, in both primary and recurrent tumors.

## Methods

### Case identification and selection

We identified all neuropathology cases with *CDKN2A* FISH analysis performed at the University of Pittsburgh Medical Center (UPMC) between 2007 and 2020 through a clinical database search. From 2007 until late 2015 *CDKN2A* FISH was performed on all primary and recurrent gliomas as part of routine clinical service, until the introduction of the GlioSeq NGS brain tumor panel in 2015. Outside of this time frame, the test was performed on a case by case basis as a part of the clinical workup at the discretion of the neuropathologist.

From this collection, we identified 151 cases of IDHm astrocytomas with *CDKN2A* FISH analysis. Beginning in 2010, *IDH* mutational status was determined during the routine diagnostic workup of new tumors by at least one of the following methods: IDH1_R132H_ immunohistochemistry, *IDH1*/*IDH2* Sanger sequencing, or *IDH1*/*IDH2* NGS (UPMC GlioSeq panel). The majority of the cohort thus consists of cases from 2010–2015, when IDH testing and *CDKN2A* FISH were concurrently in routine diagnostic use. The cohort also includes cases presenting prior to this era that were retrospectively identified as IDH-mutant based on either (a) clinical request for testing of prior material, or (b) subsequent same-site recurrence that was shown to harbor an *IDH* mutation. A single case was originally diagnosed as oligodendroglioma despite intact 1p/19q, but was reclassified as astrocytic at recurrence. All of the astrocytomas included in this study were negative for 1p/19q codeletion as determined by FISH, loss of heterozygosity (LOH) PCR, and/or NGS testing. All immunohistochemical and molecular analyses were obtained prospectively during the course of clinical service; no additional retrospective testing was performed.

The study population characteristics are shown in Table 1. From the 151 total IDHm astrocytoma specimens, we identified 108 treatment-naïve initial surgical specimens (referred to subsequently as “primary tumors” or “primary IDHm astrocytomas”). The majority of these were subtotal resections and a few were biopsies only. Clinical follow-up was available for 103 of these cases. 43 residual/recurrent resection specimens were also identified, of which 35 were first surgical recurrences (referred to subsequently as “first recurrence tumors” or “first recurrence IDHm astrocytomas”). Clinical follow-up data was available for all 35 first recurrence tumors. The majority of recurrent tumors had received some combination of chemotherapy and/or radiation therapy as part of routine clinical care. We obtained the histologic grade from the pathology electronic medical record, which was assigned according to the morphologic criteria outlined in the 2007 and 2016 revisions of the WHO Classification of Tumors of the Central Nervous System. Specifically, grade 2 tumors show minimal anaplasia and do not show significant mitotic activity, grade 3 tumors show more anaplasia and significant mitotic activity, and grade 4 tumors show mitotic activity and anaplasia with necrosis and/or endothelial proliferation. The definition of “significant mitotic activity” was applied at the discretion of the case neuropathologist, but in general at UPMC a single mitotic figure in a small biopsy or multiple mitotic figures in larger resection specimens are used as guidelines for a grade 3 designation. The single tumor that was reclassified as astrocytic after an initial diagnosis of oligodendroglioma was retrospectively given WHO grade 2 based on the absence of nuclear atypia, endothelial proliferation and necrosis, and only rare mitotic figures in a large excision specimen, in line with current diagnostic practice. We obtained clinical follow-up and mortality data through searches of the electronic medical record and/or publicly available databases as part of an Institutional Review Board approved study (IRB number: 20040135).

**Table 1:** Population characteristics of primary and first recurrence tumors

Primary tumors (*n* = 108)	Histologic grade 2 (*n* = 55)	Histologic grade 3 (*n* = 31)	Histologic grade 4 (*n* = 22)
Mean age at presentation (Years)	38.9 (19–77)	36.8 (22–79)	40.8 (23–56)
Sex (M/F)	26/29	15/16	13/9
Average length of available follow-up* (months)	65.6 (1.45–175.5)	70.5 (3.7–186.9)	44.3 (4.5–112.9)
Mean percent cells with homozygous *CDKN2A* deletion	5.00 (0–40)	8.40 (0–37.1)	23.0 (0–93.8)

First recurrence tumors (*n* = 35)	Histologic grade 2 (*n* = 8)	Histologic grade 3 (*n* = 13)	Histologic grade 4 (*n* = 14)
Mean age at recurrence	44.1 (35–58)	42 (28–64)	39.9 (28–57)
Sex (M/F)	5/3	7/6	11/3
Received adjuvant chemotherapy and/or radiation (percent)	4/8 (50%)	12/13 (92%)	14/14 (100%)
Mean duration between primary and first recurrent resection (months)	58.5 (2.5–241.6)	79.1 (12.0–184.5)	59.6 (4.8–153.1)
Average length of follow-up after recurrence (months)	65.3 (31.1–118.3)	27.6 (1.3–94.3)	18.2 (0.9–71.1)
Mean percent cells with homozygous *CDKN2A* deletion	3.2 (0–13)	24.3 (0–92.0)	53.9 (1.6–95.6)

^*^These values do not include 5 patients for whom no clinical follow-up was available

### FISH analysis

FISH for *CDKN2A* was performed on formalin-fixed paraffin embedded (FFPE) tissue sections, as previously described [13, 14]. Briefly, blank slides were deparaffinized, washed, and treated with a protease solution. The slides were then washed, and the DNA probe and tissue genomic DNA were denatured, either separately or combined depending on the time period in which the study was performed. The probe and tissue were then hybridized overnight. The UPMC lab uses the LSI 9p21/CEP9 dual color probe set for detection of *CDKN2A* deletion (Abbott Molecular, Abbott Park, IL). The 9p21 probe covers an approximately 190 kb region of 9p that includes genes *CDKN2A*, *CDKN2B*, and *MTAP*, while the CEP9 probe hybridizes to centromeric alpha satellite sequences specific to chromosome 9. The slides were then treated with post hybridization buffer, counter-stained with DAPI, and cover slipped for analysis.

Two separate histotechnologists counted and scored a minimum of 30 cells each in separate areas of a region of interest defined by the neuropathologist, for a total of 60 + cells analyzed. The histotechnologists recorded results on a grid according to the number of centromere and 9p signals present per nucleus. Results reported included percent cells with homozygous deletion, percent cells with hemizygous deletion, percent cells with complex deletion, and percent cells with hyperploidy. Homozygous deletion is defined as a cell with at least one chromosome 9 centromere signal and no 9p signals. Hemizygous deletion is defined as a cell with two chromosome 9 centromere signals and one 9p signal. Complex deletion is defined as a combination of some level of chromosome 9 centromere hyperploidy with some amount of relative loss of 9p signal.

### Data analysis

Kaplan–Meier (K-M) survival curves, multivariate Cox proportional-hazard (Cox-PH) regression analysis, Weibull accelerated failure time (AFT) models, and Akaike information criterion (AIC) analysis were performed with the Python 3 lifelines module [15]. Mantel Cox log-rank tests, distribution graphs, and a subset of K-M survival curves were created and analyzed in Prism 8.

## Results

### High percentage homozygous *CDKN2A* deletion is a rare event in primary IDHm astrocytomas, but is more frequent in recurrent tumors

**Fig. 1:**
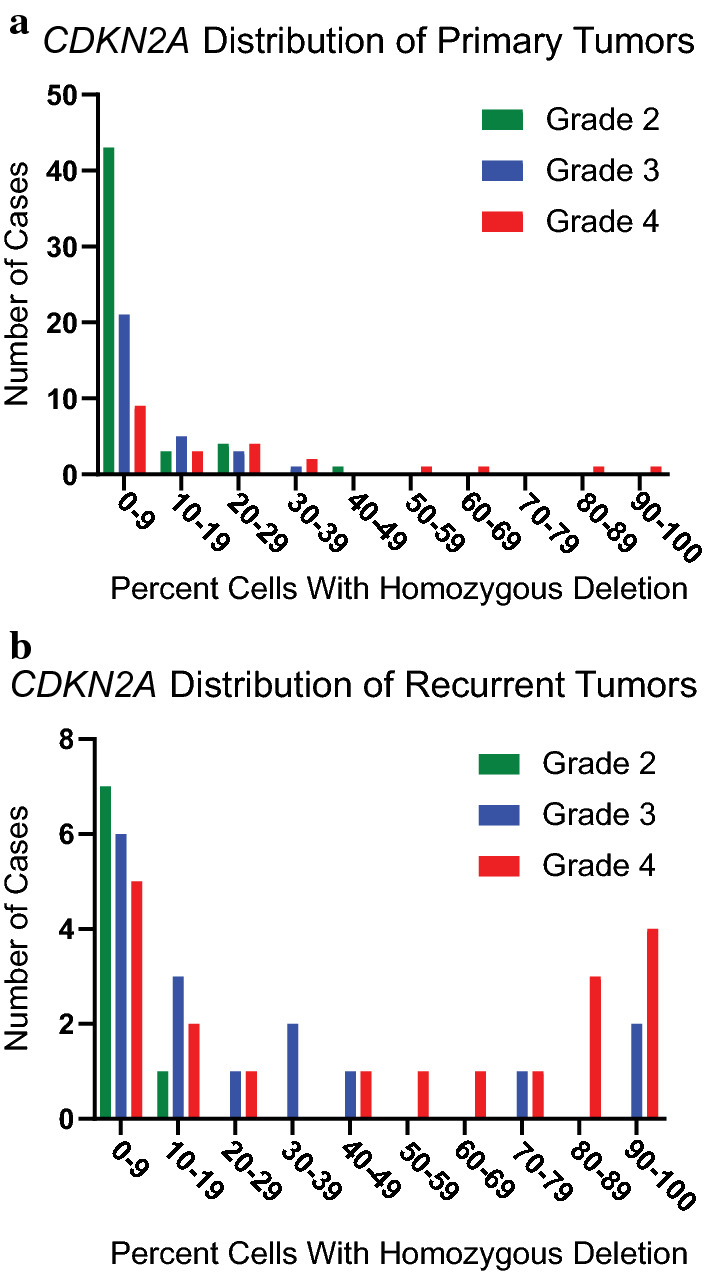
High percentage *CDKN2A* homozygous deletion is a rare event in primary IDHm astrocytomas and is more common in recurrent tumors. **a** shows the frequency distribution of the percent of tumor cells with homozygous *CDKN2A* deletion by FISH in primary IDHm astrocytomas (*n* = 108). Very few tumors have high-percentage homozygous deletion, and the majority of the tumors with high-percentage deletion are morphologic grade 4. **b** shows the frequency distribution in recurrent tumors (*n* = 43). High-percentage deletion is a more frequent event in recurrent tumors and is seen in both histologic grades 3 and 4

As a first step, we looked for evidence of a natural cut-point to distinguish tumors with intact *CDKN2A* from those with homozygous deletion, as would be seen if the percentage of cells with homozygous deletion followed a bimodal distribution. The observed distribution of percentage of cells with homozygous deletion across all primary tumors, however, was instead unimodal, with a median value less than 10% and a long right-skewed tail (Fig. 1a). Tumors of histologic grade 4 were over-represented in the tail of the distribution (including all four primary tumors with > 50% of cells having homozygous *CDKN2A* deletion), confirming a relationship between *CDKN2A* status and aggressive tumor behavior, but no intuitively obvious cutoff value emerged from this analysis.

In contrast to primary tumors, high levels of homozygous *CDKN2A* deletion occurred at a much higher relative frequency in recurrent tumor specimens (Fig. 1b). As with primary specimens, high percentage homozygous deletion mostly occurred in the setting of grade 4 histologic findings. A smaller subset of grade 3 recurrences also showed increased levels of *CDKN2A* deletion, while all tumors that continued to have grade 2 histologic features at recurrence had no or very low levels of deletion (maximum homozygous deletion among grade 2 recurrences: 13% of cells). While a few grade 4 recurrences showed essentially complete (> 90%) homozygous *CDKN2A* deletion, a substantial number also showed intermediate levels, again failing to define an obvious cutoff value.

**Fig. 2:**
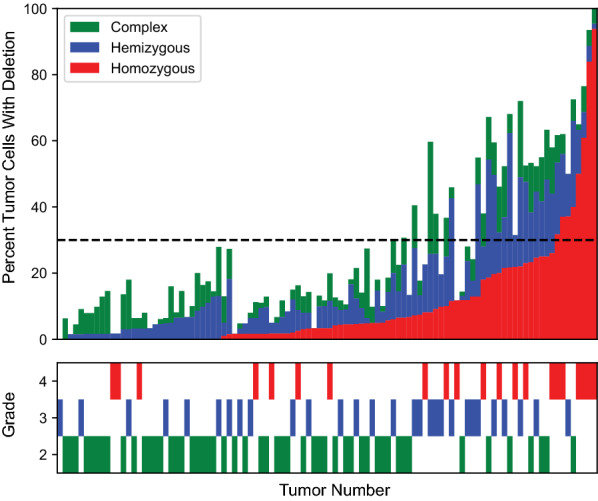
*CDKN2A* homozygous deletion, hemizygous loss, and complex loss in primary tumors. The per-tumor distribution of *CDKN2A* homozygous deletion, hemizygous loss, and complex loss is shown for primary IDHm astrocytomas for which all data was available (*n* = 102). Each column represents the distribution of loss within an individual tumor. Tumors are ordered by percent homozygous deletion, then percent hemizygous loss, and finally percent complex loss. The bottom graph corresponds to each above tumor’s histologic grade

Finally, we examined the relationship between hemizygous loss, homozygous deletion, and complex loss in primary IDHm astrocytomas (Fig. 2). These findings must be interpreted with some caution as hemizygous and complex loss events are subject to increased rates of technical artifact due to partially-sectioned nuclei resulting in artificial loss of signal, an intrinsic characteristic of FISH performed on fixed paraffin embedded tissue sections. A subset of tumors shows significantly higher levels of hemizygous and/or complex loss in comparison to homozygous deletion. The significance of this finding is not clear, but it may represent tumor evolution toward homozygous deletion.

### A cutoff of ≥ 30% homozygous deletion by FISH maximizes the prognostic value of *CDKN2A* in IDHm astrocytomas

**Fig. 3:**
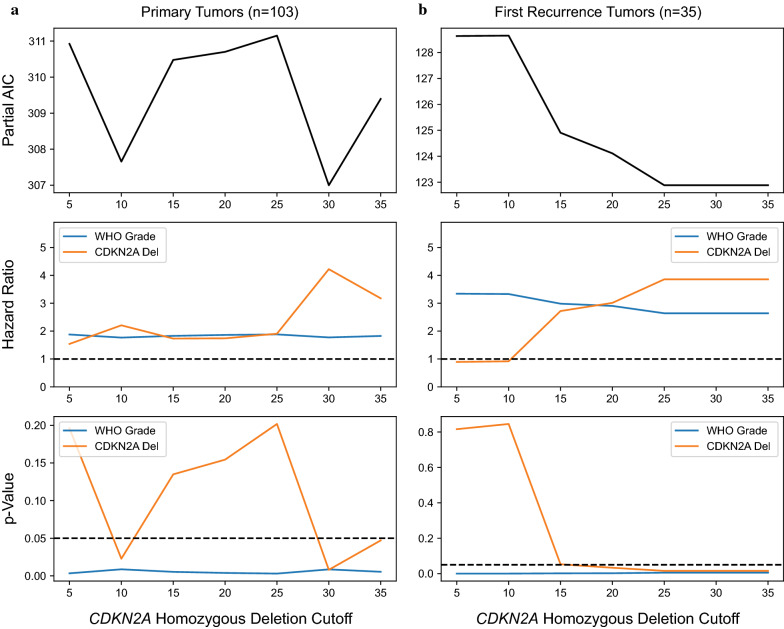
A cutoff of ≥ 30% homozygous deletion by FISH maximizes the prognostic value of *CDKN2A* in IDHm astrocytomas. Cox-PH models were constructed using a range of *CDKN2A* cutoff values from 5 to 35%, as well as sex, age, and histologic grade. At each cutoff, the partial AIC value, hazard ratios, and p-values of the model fit for each parameter were assessed. Models were constructed independently for primary tumors (**a**) and first recurrences (**b**). For primary tumors, the hazard ratio (middle row, orange line) and statistical significance (bottom row, orange line) of *CDKN2A* deletion are maximized and the AIC (top row, black line) is minimized at a cutoff of ≥ 30%, indicating optimal model characteristics. For first recurrence tumors, cutoffs between 25–35% perform equally across the three measures. Varying the *CDKN2A* cutoff does not substantially influence the hazard ratio or significance of histologic grade as an independent prognostic variable (middle and bottom rows, blue lines)

Using a multivariate Cox-PH model including patient age, sex, histologic grade, and percent *CDKN2A* homozygous deletion as a continuous variable, we verified that histologic grade and *CDKN2A* deletion were independently significant prognostic factors in our cohort (Additional file 1: table S1). From a practical standpoint, however, deciding whether an individual tumor specimen is “positive for *CDKN2A* homozygous deletion” for grading purposes requires a defined cutoff value for the percentage of cells with homozygous deletion by FISH. To determine a clinically appropriate cutoff, we constructed series of Cox-PH models as above, but with *CDKN2A* status binarized at cutoff thresholds ranging from 5–35% cells with homozygous deletion (Fig. 3a, Additional file 1: table S2). In our cohort of primary tumors, a cutoff of ≥ 30% homozygous deletion minimized the AIC of the model fit, minimized the Cox-PH p-value, and maximized the hazard ratio for *CDKN2A* loss (Fig. 3a). We repeated this analysis for the first recurrence tumors (Fig. 3b, Additional file 1: table S3). In these specimens, cutoff values between 25 and 35% performed equally across the range. Based on these data, we selected ≥ 30% homozygous deletion by FISH as a rational cutoff for defining *CDKN2A* homozygous deletion for the subsequent survival analyses.

### Both *CDKN2A* homozygous deletion and histologic grade are independent predictors of survival in primary and first recurrence IDHm astrocytomas

**Fig. 4:**
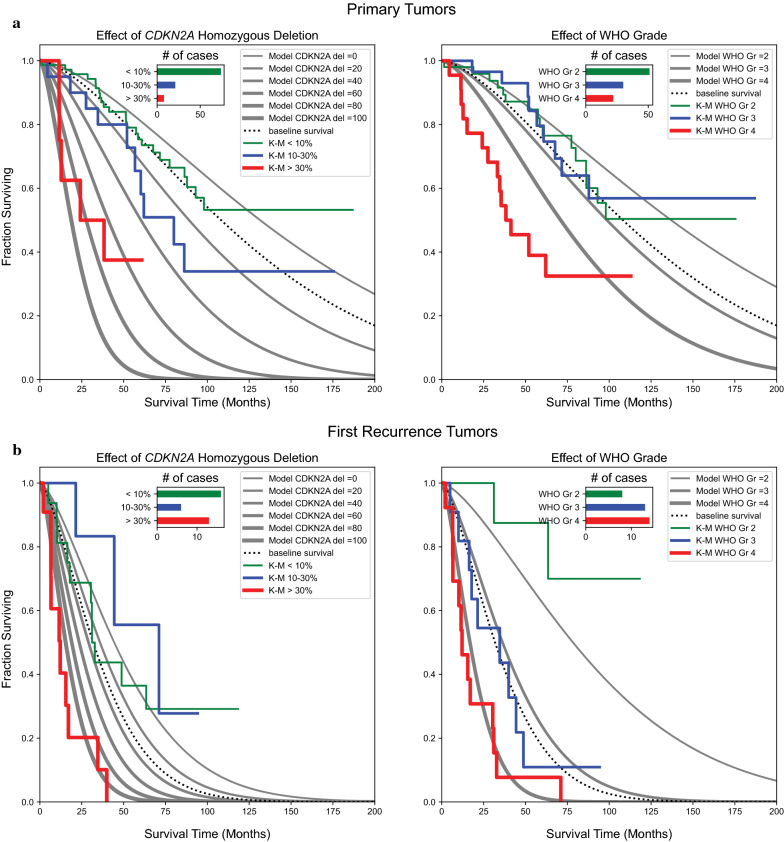
Both *CDKN2A* homozygous loss and histologic grade are independent predictors of survival in primary and first recurrence IDHm astrocytomas**.** K–M survival curves (colored lines) and Weibull AFT models (gray lines) for primary tumors (**a**) and first recurrences (**b**), stratified by percent homozygous *CDKN2A* deletion (left) and WHO histologic grade (right). For primary tumors, ≥ 30% *CDKN2A* homozygous deletion is associated with significantly shorter survivals than < 30% loss. While there was no separation between histologic grades 2 and 3, histologic grade 4 tumors showed significantly shorter survival than grades 2 and 3 combined. For first recurrence tumors, ≥ 30% *CDKN2A* deletion is similarly associated with shorter overall survival. By morphologic grading, survival of recurrent grade 3 tumors tracked closely with recurrent grade 4 tumors, both of which were significantly shorter than grade 2 tumors. The observed survival functions (K-M curves) closely track the parametric Weibull AFT model which separates the effects of *CDKN2A* deletion and histologic grade

Both *CDKN2A* homozygous deletion ≥ 30% (*p* = 0.01, HR = 4.22) and histologic grade (*p* = 0.01, HR = 1.77) emerged as independent predictors of outcome for primary tumors in our Cox-PH model (Additional file 1: table S2). The K-M survival curves and associated Weibull AFT models for primary IDHm astrocytomas stratified solely by *CDKN2A* homozygous deletion or histologic grade are shown in Fig. 4a. When stratified by *CDKN2A* status, primary tumors with ≥ 30% homozygous deletion had significantly worse survival than those with < 30% deletion (p = 0.0009, log-rank test). Tumors with mid-level deletion (10–30%) fared somewhat worse than those with low level deletion (< 10%), but this difference was not significant (*p* = 0.0636, log-rank test). Using the 2007/2016 WHO Classification histologic grading criteria, there was minimal to no separation of grade 2 and grade 3 tumors, as has been previously demonstrated by multiple groups [3, 4, 11]. Primary histologic grade 4 tumors had significantly decreased survival compared to histologic grades 2/3 (*p* < 0.0001, log-rank test).

The above analyses were repeated for the first recurrence tumors. Again, both *CDKN2A* (*p* = 0.02, HR = 3.86) and histologic grade (*p* = 0.01, HR = 2.64) were independent predictors of outcome (Additional file 1: table S3). The survival curves from the date of re-resection stratified by *CDKN2A* and histologic grade alone are shown in Fig. 4b. Tumors with low (< 10%) and mid-level (10–30%) *CDKN2A* deletion showed similar survivals, while tumors with ≥ 30% deletion had significantly decreased survival (*p* = 0.0003, log-rank test). Interestingly, morphologic grade 3 recurrent tumors fared significantly worse than their primary grade 3 counterparts, tracking closely with recurrent grade 4 tumors in our cohort. Tumors that were grade 2 on recurrence had significantly longer overall survival compared to tumors that were grade 3 or grade 4 on recurrence (*p* = 0.0064 for grade 3, *p* = 0.0001 for grade 4, log-rank tests).

### *CDKN2A* deletion by FISH stratifies survival of grade 4 tumors, but not lower grades

**Fig. 5:**
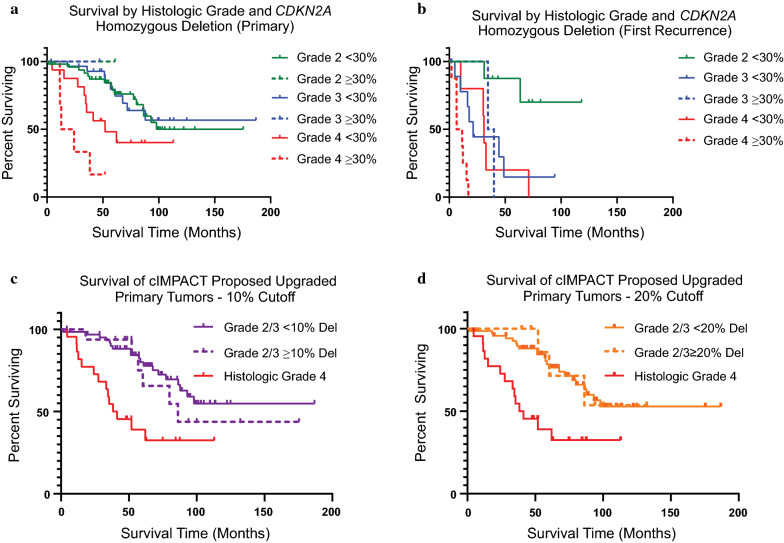
*CDKN2A* deletion by FISH stratifies survival for grade 4 tumors, but not lower grades. K-M survival curves for tumors above and below 30% homozygous *CDKN2A* deletion at each histologic grade are shown for primary tumors (**a**) and first recurrence tumors (**b**). In primary tumors, only 2 of 86 grade 2/3 tumors showed ≥ 30% *CDKN2A* homozygous deletion, and both patients were still living at the time of analysis. 6 of 22 primary grade 4 tumors showed ≥ 30% deletion, and patients with these tumors had significantly shorter survival than grade 4 tumors without loss. Among recurrences, no grade 2 tumors showed ≥ 30% deletion. Recurrent grade 3 tumors with and without *CDKN2A* deletion showed similar survival times. As with primary tumors, grade 4 recurrences with *CDKN2A* deletion showed significantly decreased survival compared to those grade 4 tumors without deletion. Survival curves for tumors that would hypothetically be upgraded at lower *CDKN2A* homozygous deletion cutoffs of 10% (**c**) or 20% (5D) are shown in relation to histologic grade 4 tumors. Although more tumors are identified as having *CDKN2A* deletion at these cutoff values, meaningful improvements in prognostic stratification are not seen

Finally, we assessed the potential impact of incorporating *CDKN2A* status into tumor grading criteria in our institutional cohort. We began by constructing K-M survival curves of primary IDHm astrocytomas, stratified both by WHO histologic grade and *CDKN2A* deletion (Fig. 5a). Unlike the previously published studies using array-based data [7, 10], FISH-based detection of *CDKN2A* deletion of ≥ 30% did not identify a subset of histologically low-grade tumors with highly aggressive behavior. Only a single grade 2 tumor and a single grade 3 tumor in our cohort would be upgraded under Consortium to Inform Molecular and Practical Approaches to CNS Tumor Taxonomy – Not Official WHO (cIMPACT-NOW) 5/6 criteria [3, 12] using a ≥ 30% cutoff, and both patients were still living at the time of analysis, with survival durations of 61 and 47 months, respectively. For comparison, the median survival of all histologic grade 4 tumors in our cohort was 40 months. Grade 4 tumors with *CDKN2A* homozygous deletion had significantly shorter survival than those without loss (p = 0.032, log-rank test).

The above analyses were repeated for first recurrence tumors (Fig. 5b). No recurrent grade 2 tumors showed significant *CDKN2A* homozygous deletion. There was no difference in survival in recurrent grade 3 tumors with deletion compared to those without deletion (*p* = 0.837, log-rank test), and all histologic grade 3 recurrences showed similar survival to histology grade 4 tumors without deletion. Grade 4 recurrent tumors with *CDKN2A* homozygous deletion showed significantly shorter overall survival than grade 4 tumors without deletion (p = 0.0079, log-rank test).

**Fig. 6:**
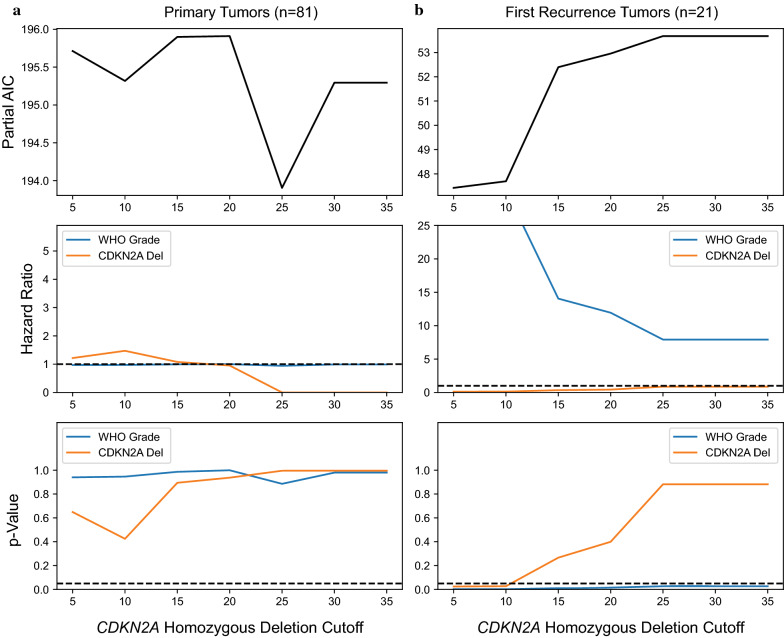
Cox-PH model and AIC analysis restricted to lower grade tumors fails to identify an appropriate *CDKN2A* cutoff. For primary grade 2/3 tumors, neither *CDKN2A* homozygous deletion (**a**, orange line) nor histologic grade (blue line) were significant predictors of outcome across the range of tested values (HR ≤ 1, *p *values > 0.05). *CDKN2A* deletion is also not a significant predictor in grade 2/3 first recurrence tumors (**b**, HR ≤ 1, *p *values > 0.05), while histologic grade is significant in this cohort

In order to address the possibility that a ≥ 30% cutoff in primary IDHm astrocytomas might be too stringent, we performed additional K-M survival analysis on grade 2/3 tumors that would hypothetically be upgraded to grade 4 under the proposed cIMPACT-NOW 5/6 criteria using *CDKN2A* homozygous deletion thresholds of 10% and 20% tumor cells deleted (Fig. 5c and d). Using a 10% cutoff, 8 grade 2 tumors and 9 grade 3 tumors would be upgraded. Using a 20% cutoff, 5 grade 2 tumors and 4 grade 3 tumors would be upgraded. At both of these thresholds, the potentially upgraded tumors showed longer survival than histologic grade 4 tumors, a finding which reached statistical significance despite the small sample size (*p* = 0.036 for 10%, *p* = 0.0499 for 20%, log-rank test). The magnitude of the difference was also clinically meaningful. Lower grade tumors subject to upgrading based on a 10% *CDKN2A* cutoff had a median survival advantage of 46 months compared to histologic grade 4 tumors; median survival was not reached in tumors upgraded using 20% or greater cutoffs. Finally, we repeated our Cox-PH analysis, restricting to only grade 2 and grade 3 primary or first recurrence tumors (Fig. 6). For both primary and first recurrence non-grade 4 tumors, *CDKN2A* homozygous deletion was not prognostically significant across the range of tested values.

## Discussion

Grading of diffuse gliomas is a critical component of the diagnostic process that guides therapeutic decision-making and should, ideally, be based on prognostically significant measures of tumor behavior. Based on recently published recommendations by the cIMPACT-NOW working group [3, 12], *CDKN2A* status is likely to be incorporated into future grading systems for IDHm astrocytomas, but it remains unclear how to interpret the results of a commonly used testing modality, FISH, for this purpose. In order to define an appropriate cutoff value for interpreting *CDKN2A* FISH results as positive for homozygous deletion in routine clinical service, and to assess the anticipated impact of applying these results in our patient population using the proposed cIMPACT-NOW grading schemes, we performed a retrospective analysis of IDHm astrocytomas that had prospectively undergone *CDKN2A* FISH analysis. Multivariate Cox-PH analysis revealed that the prognostic significance of *CDKN2A* in IDHm astrocytomas is optimized by using a cutoff value of ≥ 30% of tumor cells with homozygous deletion in order to define tumor-wide “homozygous deletion” status. Applying this cutoff value, very few primary treatment-naïve tumors in our institutional cohort showed this level of deletion, and of the tumors that did exceed this threshold, the majority were already considered grade 4 by histology. Compared to primary tumors, *CDKN2A* homozygous loss was more frequent in recurrent grade 3 and grade 4 tumors. While both histologic grade and *CDKN2A* loss were independent predictors of survival in our cohort, the inclusion of *CDKN2A* as a grading criterion failed to improve survival stratification, at least in the setting of tumors not meeting grade 4 criteria histologically. With the ≥ 30% cutoff, only two lower grade tumors would be upgraded to grade 4, and both patients were still living at the time of analysis, having already exceeded the median survival of histologic grade 4 tumors.

At first blush, it may seem counterintuitive that the Cox-PH model developed using the full cohort of tumors spanning all grades confirms *CDKN2A* deletion as an independently significant prognostic factor, yet the optimal cutoff of 30% was exceeded by only a very small fraction of grade 2 and 3 tumors. These findings can be reconciled, however, if the overall model is being largely driven by prognostic differences among morphologically grade 4 tumors. Indeed, unlike lower grade tumors, grade 4 tumors with ≥ 30% *CDKN2A* deletion showed significantly shorter overall survival than grade 4 tumors with intact *CDKN2A*, in both primary and first recurrence tumors (Fig. 5a and b). In light of the published studies claiming dismal prognosis for lower grade tumors with *CDKN2A* loss [7, 10, 11], we wanted to make sure that the model driven by grade 4 effects was not inappropriately excluding certain grade 2–3 tumors from being classified as a higher molecular grade; perhaps a different, lower threshold might be more appropriate for stratifying the lower grade tumors. We addressed this question in two ways: by applying a lower threshold and examining whether hypothetically-upgraded tumors behave as the adjusted grade would suggest (Fig. 5c and d) and by creating a Cox-PH model using only grade 2 and 3 tumors (excluding grade 4) (Fig. 6). Contrary to the hypothesis that a lower threshold would perform better for lower grade tumors, both analyses show the opposite—in our cohort of patients, there is no evidence that a threshold at or below 30% homozygous deletion improves prognostication of lower-grade lesions. We note, however, that it remains possible that lower grade IDHm astrocytomas with high level (≥ 30%) *CDKN2A* deletion by FISH do in fact have a poor prognosis, but given the rarity of grade 2 or 3 tumors exceeding this threshold, there is insufficient evidence to determine the prognostic impact of this finding in our cohort.

Our findings differ from those studies showing a significant prognostic impact of *CDKN2A* deletion in both grade 3 and grade 4 IDHm astrocytomas, as detected by array-based techniques [7, 10]. While our data show a similar poor prognosis of *CDKN2A* deletion in grade 4 IDHm astrocytomas, these findings do not extend to grade 3 tumors. Using the proposed cIMPACT-NOW 5/6 criteria and our statistically-defined FISH cutoff of ≥ 30%, the hypothetical upgrade rate of grade 3 tumors in our patient cohort is markedly lower (1/31, 3%) than that in Shirahat et al*.* (15/90, 17%) or Appay et al*.* (35/211, 17%). While it is possible that technical differences between various testing modalities could have an unexpectedly large effect, a likelier cause of this discrepancy could be differences in the population characteristics of the tumors being analyzed. The histologic grades at presentation for tumors in the cohorts of Shirahat et al*.* (26% grade 2, 43% grade 3, 32% grade 4) and Appay et al*.* (grade 2 not included in primary analysis, 50% grade 3, 50% grade 4) are skewed towards higher grades, while our single institution cohort had a larger proportion of lower grade tumors (51% grade 2, 29% grade 3, and 20% grade 4). The distribution of cases in our study, with the highest proportion presenting as grade 2 and the lowest as grade 4, is in broad agreement with previously published large all-comer cohorts of IDHm astrocytomas [4]. Similarly, the frequency at which homozygous *CDKN2A* loss was detected in our cohort at each grade (grade 2: 1.8%, grade 3: 3.2%, grade 4: 27%) is similar to the proportion of IHDm astrocytomas in The Cancer Genome Atlas (TCGA) combined low grade glioma and glioblastoma cohorts (grade 2: 3.5%, grade 3: 6.7%, grade 4: 18.8%; primary tumors, multiple methods of *CDKN2A* assessment) [8]. The enrichment in grade 3 and grade 4 tumors in the Shirahat et al. and Appay et al. studies, along with the elevated frequency of *CDKN2A* loss in grade 3, suggests that these cohorts may represent a different population of tumors, possibly including increased numbers of diagnostically difficult, borderline, or clinically aggressive cases sent for expert consultation. It is also not clear whether these studies limited their analyses to only primary treatment-naïve specimens. Inclusion of recurrent/treated tumors could be a significant confounder, especially given the overall worse prognosis and increased frequency of *CDKN2A* loss seen in recurrent tumors in our cohort.

The existing literature on use of FISH specifically to detect *CDKN2A* homozygous deletion in gliomas is sparse. Perhaps the most relevant is a recent study by Yang et al*.* that used FISH to examine *CDKN2A, CDK4,* and *PDGFRA* copy number alterations in grade 2 and 3 astrocytomas [11]. The authors used a cutoff of ≥ 20% of tumor cells showing homozygous deletion, but a rationale for this threshold was not provided. The study found *CDKN2A* homozygous deletion of ≥ 20% at similar rate in grade 2/3 tumors as our cohort (15% Yang et al*.* versus 10% for our cohort) with both grade 2 and grade 3 tumors showing deletion. The survival effect of *CDKN2A* in the Yang et al*.* cohort cannot be directly evaluated, as the survival analysis in that study grouped tumors with *CDKN2A* deletion with those showing *CDK4* amplification (i.e. alteration in the RB1 pathway), rather than assessing each gene independently. While these RB1-altered tumors did show shorter overall survival than the RB1-intact group, they nonetheless appear to have a significantly better prognosis than the morphologic grade 4 tumors in our study. This comparison with the published literature further supports our findings that grade 2/3 astrocytomas with *CDKN2A* homozygous deletion as assessed by FISH have longer survival than histologic grade 4 tumors.

The ability to accurately and reliably detect *CDKN2A* homozygous deletion in IDHm astrocytomas is necessary if this is to be included as a grade defining criteria in the next revision of the WHO Classification. The cIMPACT-NOW recommendations do not offer definitive guidance as to which testing modalities should be used, and there are many possible methods to detect loss. FISH is perhaps the oldest and most widely accepted technique for detecting copy number alterations. FISH testing has many benefits, including the ability to definitively identify homozygous deletion in infiltrating tumors, and to separate true homozygous deletion from hemizygous loss and complex copy number alterations. Like all testing modalities, however, FISH has certain intrinsic technical limitations, including insensitivity to deletions smaller than the region covered by the probe, and artifactual loss of signal due to partial sectioning of nuclei when performed on FFPE tissue sections. As an example of the latter, consider a nucleus with a single CEP9 signal and no 9p21 signals. This result could accurately reflect monosomy 9 with additional 9p21 deletion, but could also arise in other ways: (1) homozygous 9p21 deletion with artifactual loss of one CEP9 signal; (2) monosomy 9 with artifactual loss of one 9p21 signal; (3) hemizygous 9p21 loss paired with artifactual loss of one CEP9 and one 9p21 signal; (4) wild type chromosome 9 with artifactual loss of three probes. The possibility of artifactual signal loss highlights the necessity of interpretative guidelines. In our laboratory, a nucleus with no 9p21 signals and at least one CEP9 signal is interpreted as homozygous deletion, which favors sensitivity for detecting true absence of *CDKN2A* over specificity for excluding monosomy 9 with artifactual 9p21 loss. A more stringent criteria requiring two CEP9 signals would lead to even fewer tumors being identified as having homozygous *CDKN2A* deletion.

In addition to FISH, there are numerous variations of genomic microarrays in use for analyzing brain tumor samples. Comparative genomic hybridization arrays (aCGH) excel at detecting copy number alterations in aggregate tissue samples, but can struggle at detecting loss in the context of a sparsely infiltrating tumor. Single nucleotide polymorphism (SNP) arrays are often used in combination with either true or virtual aCGH to help separate hemizygous loss and complex alteration events from true homozygous loss (although not at a single cell level). Finally, the data obtained from methylation or other NGS arrays can often be analyzed to provide output similar to a combination aCGH/SNP array. As with FISH, each of these techniques have their own interpretive subtleties and technical limitations, which complicates cross-modality comparisons of the prognostic implications of test results. Clear and specific descriptions of the algorithms used to judge homozygous deletion status would be helpful for this purpose, but are frequently lacking. As a result, direct comparison between these various methods is often not possible, and it is not clear which technique should represent the “gold standard.”

An important point of concordance between our study and the existing literature is the lack of prognostically meaningful *CDKN2A* homozygous deletion in histologically grade 2 tumors. The lone patient with a grade 2 tumor with over 30% homozygous deletion in our study is still alive at 61 months from diagnosis, and neither of the above array-based studies identified any histologic grade 2 tumors with *CDKN2A* homozygous deletion (Shirahat et al*.* *n* = 54, Appay et al*.* *n* = 20). The wording of the cIMPACT-NOW 6 proposal for updated grading criteria is not clear regarding whether or not morphologic grade 2 IDHm tumors will require *CDKN2A* testing for formal grading; clarification on this point will be essential in any upcoming WHO update. Results from our cohort agree with the previous studies and suggest that testing grade 2 tumors for deletion would be very low yield at best, might lead to inappropriate upgrading of indolent tumors, and could impose an undue financial burden on the global healthcare system.

In conclusion, *CDKN2A* homozygous deletion is a marker of poor prognosis in histologic grade 4 IDHm astrocytomas, but the impact of this finding in histologic grades 2 and 3 tumors is less clear. Different techniques for determining *CDKN2A* status may provide markedly different results between and even within individual institutions. Specific criteria for determining the presence of homozygous deletion across different testing modalities will be essential if *CDKN2A* homozygous deletion is included as a grading criterion in the next revision of the WHO Classification.

## Supplementary information

**Additional file1** Full details of the Cox Proportional Hazards models used in this study.

## Data Availability

The datasets compiled and analyzed during the current study are available in deidentified form from the corresponding author upon reasonable request.
