# Supplementary material for: Homozygous deletion of CDKN2A by fluorescence in situ hybridization is prognostic in grade 4, but not grade 2 or 3, IDH-mutant astrocytomas
Source: Acta Neuropathol Commun. 2020 Oct 20;8:169. doi: 10.1186/s40478-020-01044-y (PMC7574334; doi:10.1186/s40478-020-01044-y)
Supplement: Supplementary file 1 — Additional file1 Full details of the Cox Proportional Hazards models used in this study. [file 40478_2020_1044_MOESM1_ESM.pdf]

Supplementary Table S1: Cox Proportional Hazard Model for Primary Tumors with *CDKN2A* Loss as a Continuous Variable

Hazard ratios:

Female Sex	0.524919
WHO Grade	1.661930
Age at Resection	1.028102
CDKN2A Homo Del	1.031800

	coef	exp(coef)	se(coef)	coef lower 95%	coef upper 95%	exp(coef) lower 95%	exp(coef) upper 95%	z	p	-log2(p)
**Sex**	-0.64	0.52	0.34	-1.31	0.02	0.27	1.02	-1.89	0.06	4.10
**Grade**	0.51	1.66	0.23	0.07	0.95	1.07	2.59	2.25	0.02	5.37
**Age**	0.03	1.03	0.01	0.00	0.05	1.00	1.06	2.07	0.04	4.71
**CDKN2A Homo Del**	0.03	1.03	0.01	0.01	0.05	1.01	1.05	3.17	<0.005	9.35

**Concordance**	0.69
**Partial AIC**	304.18
**log-likelihood ratio test**	23.13 on 4 df
**-log2(p) of ll-ratio test**	13.03

Supplementary Table S2: Cox Proportional Hazard Model for Primary Tumors with ≥30% Cutoff

Hazard ratios:

Female Sex	0.535655
WHO Grade	1.773531
Age at Resection	1.030576
CDKN2A Homo Del	4.220045

	coef	exp(coef)	se(coef)	coef lower 95%	coef upper 95%	exp(coef) lower 95%	exp(coef) upper 95%	z	p	-log2(p)
**Sex**	-0.62	0.54	0.34	-1.29	0.04	0.28	1.04	-1.85	0.06	3.95
**Grade**	0.57	1.77	0.22	0.15	1.00	1.16	2.72	2.63	0.01	6.86
**Age**	0.03	1.03	0.01	0.00	0.06	1.00	1.06	2.24	0.02	5.33
**CDKN2A Homo Del ≥30%**	1.44	4.22	0.54	0.37	2.51	1.45	12.25	2.65	0.01	6.95

**Concordance**	0.70
**Partial AIC**	307.00
**log-likelihood ratio test**	20.31 on 4 df
**-log2(p) of ll-ratio test**	11.17

Supplementary Table S3: Cox Proportional Hazard Model for First Recurrence Tumors with ≥30% Cutoff

Hazard ratios:

Female Sex	0.605481
WHO Grade	2.638756
Age at Resection	0.995928
CDKN2A Homo Del	3.855558

	coef	exp(coef)	se(coef)	coef lower 95%	coef upper 95%	exp(coef) lower 95%	exp(coef) upper 95%	z	p	-log2(p)
**Sex**	-0.50	0.61	0.53	-1.53	0.53	0.22	1.70	-0.95	0.34	1.56
**Grade**	0.97	2.64	0.35	0.29	1.66	1.33	5.23	2.78	0.01	7.51
**Age**	-0.00	1.00	0.02	-0.05	0.04	0.96	1.04	-0.19	0.85	0.24
**CDKN2A Homo Del ≥30%**	1.35	3.86	0.56	0.26	2.44	1.29	11.51	2.42	0.02	6.00

**Concordance**	0.80
**Partial AIC**	122.88
**log-likelihood ratio test**	23.36 on 4 df
**-log2(p) of ll-ratio test**	13.19
